# Medication adherence and complementary therapy usage in inflammatory bowel disease patients during the coronavirus disease 2019 pandemic

**DOI:** 10.1002/jgh3.12537

**Published:** 2021-03-29

**Authors:** Alex Barnes, Jane Andrews, Paul Spizzo, Réme Mountifield

**Affiliations:** ^1^ Department of Gastroenterology Flinders Medical Centre Bedford Park South Australia Australia; ^2^ College of Medicine and Public Health Flinders University Bedford Park South Australia Australia; ^3^ Inflammatory Bowel Disease Service, Department of Gastroenterology and Hepatology Royal Adelaide Hospital Adelaide South Australia Australia; ^4^ Faculty of Health & Medical Sciences, School of Medicine University of Adelaide Adelaide South Australia Australia

**Keywords:** COVID‐19, gastroenterology, immunology, medication adherence, microbiology and inflammatory bowel diseases

## Abstract

**Background and Aim:**

Medication nonadherence is common in patients with inflammatory bowel disease (IBD) and has been associated with worse outcomes. The coronavirus disease 2019 (COVID‐19) pandemic led to significant consumer and medical concern regarding the possible risks of immunosuppressive medications during the pandemic. This study aimed to examine medication adherence and complementary and alternative medicine (CAM) usage during the COVID‐19 pandemic.

**Methods:**

An online survey was sent to patients from two tertiary IBD units. The survey included medication nonadherence attributed to the COVID‐19 pandemic, complementary therapy, and IBD medication use. Validated measures of IBD disease activity, medication adherence, and beliefs about medicines were obtained.

**Results:**

Of 262 respondents (median age of 46, 58% female) 14 (5%) patients reported self‐initiated missed doses or dose reduction of IBD medications directly attributed to the COVID‐19 pandemic. Positive associations with medication nonadherence included current corticosteroid requirement (*P* = 0.022), higher disease activity scores (*P* = 0.026), and higher concern about medicines score (*P* = 0.04). CAM usage was common, aimed at treating mental health in most cases, and infrequently attributed to the COVID‐19 pandemic.

**Conclusions:**

Even in the setting of low COVID‐19 prevalence, the pandemic reduced IBD medication adherence in 1 in 20 patients. This reduced adherence was co‐associated with increased disease activity and corticosteroid use. Understanding the underlying beliefs driving suboptimal IBD medication adherence is critical to prevent avoidable adverse IBD outcomes.

## Graphical abstract

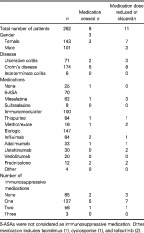

The coronavirus disease 2019 (COVID‐19) pandemic resulted in widespread concern that inflammatory bowel disease (IBD) medications may result in higher infection risk and poor outcomes. The rate of medication nonadherence attributed to the COVID‐19 pandemic in an Australian IBD population was low and associated with increased IBD disease activity, corticosteroid use, and patient concern over medications. Understanding the underlying beliefs driving suboptimal IBD medication adherence is critical to prevent avoidable adverse IBD outcomes.

## Introduction

The pandemic caused by the novel coronavirus severe acute respiratory syndrome coronavirus 2 (SARS‐CoV‐2) has resulted in widespread fear, and dramatically influenced governmental and individual behaviors. At particular risk of adverse outcomes from infection are those of advanced age, and those with comorbidities.^1^ During the early stages of the SARS‐Cov‐2 pandemic, there was considerable concern in both medical and patient inflammatory bowel disease (IBD) communities that immunosuppressive IBD medications may result in greater SARS‐CoV‐2 infection rates and poorer outcomes.^2^ Evidence to support this negative effect is lacking however,^3^ and in contrast it has been speculated that some IBD medications may have a protective effect against the cytokine storm seen in severe coronavirus disease 2019 (COVID‐19).^4^ In Australia as around the world, Gastroenterology societies hastily distributed information, advising people with IBD to remain on their medications unless they develop COVID‐19,^5^, ^6^ while acknowledging the uncertainty and lack of robust data on which these recommendations were based.

Medication nonadherence in IBD has been found to be associated with higher disability, poor quality of life, higher morbidity and mortality, and increased disease activity or relapse.^7^, ^8^, ^9^ Complementary and alternative medicine (CAM) usage is common in patients with IBD, with an Australian cohort reporting a prevalence of 45%^10^ and has, relevantly to the COVID‐19 pandemic, been associated with medication nonadherence.^11^ Reasons for CAM use in an IBD population have been reported as the desire for greater control, the perceived favorable safety profile, and the lack of efficacy of conventional medical therapy.^12^, ^13^, ^14^

The beliefs about medicines questionnaire (BMQ) was developed to understand the medication‐related beliefs of people with chronic illnesses.^15^, ^16^ Necessity, as measured by the BMQ, has been associated with adherence in non‐IBD populations.^17^ Similarly, greater levels of medication concern as measured by the BMQ have been associated with nonadherence.^17^ The BMQ is a tool that can be used to evaluate people's medication beliefs and may in part aid in understanding nonadherence during the COVID‐19 pandemic.

Anecdotally, our tertiary IBD centers received many enquiries from concerned patients keen to cease or dose reduce their medications early in the COVID‐19 pandemic. However, there are limited published data to objectify this observation regarding IBD patient perceptions and behavior during the COVID‐19 pandemic.

The aim of this study was to explore medication adherence and complementary therapy usage during the COVID‐19 pandemic, and examine the beliefs underlying medication de‐adherence in this setting.

## Methods

An anonymous online survey of people with IBD was undertaken, using the email databases from the IBD units from two tertiary centers in South Australia. The survey included basic demographics, disease‐related data, and current IBD medication use (see Appendix S2). The medication adherence rating scale (MARS) and the BMQ were used. A novel complementary medication question and COVID‐19 pandemic medication survey were used (see Appendix S1).

### COVID‐19 pandemic

Respondents were asked about their IBD medications during the COVID‐19 pandemic (see Appendix S1), on whose advice were changes to their medication made, and whether changes were directly attributable to the COVID‐19 pandemic. They were then asked to provide specifics regarding which medications were changed and in what manner.

### Complementary and alternative medicines

Respondents were asked which complementary therapies they used, the condition intended to treat including CAM use as a result of the COVID‐19 pandemic, perceived effectiveness, and level of consultation, if any, with conventional doctor or pharmacist. A five‐point Likert scale was used to assess the frequency and perceived effectiveness of complementary therapies, whereby ‘Frequent complementary therapy usage’ was defined as at least sometimes use.

### Disease activity

Patient‐reported disease activity was assessed. Patients with ulcerative colitis or indeterminate colitis (IBD‐U) completed the Simple Clinical Colitis Activity Index (SCCAI),^18^ with clinical remission defined as an SCCAI of less than 5.^18^ Patients with Crohn's disease were evaluated with the Harvey Bradshaw Index (HBI),^19^ with clinical remission defined as a HBI less than 5.^19^

### Medication adherence rating scale

Medication adherence was assessed using a validated medication adherence score—the MARS.^20^ The MARS is a 10‐item self‐reported survey that has combined elements from the Medication adherence questionnaire and the Drug Attitude inventory. Item results are weighted and added to provide a score that allows classification into non‐adherent, partially adherent, or adherent.

### Beliefs about medicines questionnaire

The BMQ is a validated questionnaire that assesses personal beliefs around medications including concern, necessity, overuse, and harm.^15^ The BMQ has been validated in various chronic diseases including IBD, rheumatoid arthritis, heart disease, and asthma.^15^, ^17^ The Necessity score assesses the perceived necessity of the prescribed medication. The Concern score assesses concerns about potential adverse effects from the prescribed medication. A necessity–concerns differential is calculated as the difference between the Necessity and the Concerns scales.

Ethics approval was granted from the Southern Adelaide Human Research Ethics Committee. Patients were included if they completed the COVID‐19 pandemic portion of the questionnaire. One missing answer in any of the scores such as HBI, SCCAI, BMQ, or MARS resulted in it being excluded from subsequent analysis. All statistical analysis was performed using Stata version 15 (Statacorp LLC, College Station, Texas, USA). For normally distributed variables, mean and SD were reported with comparisons made using the Student's *t*‐test. For non‐normally distributed variables, median and interquartile range (IQR) were reported, with comparisons made using the Wilcoxon rank‐sum test. For categorical data, Pearson's *χ* ^2^ test was used, or Fisher's exact test when appropriate.

## Results

There were 262 respondents from 861 emailed survey requests (30.3% interest in survey, 87.0% survey completion rate) over the time period May 2020 to July 2020. The local COVID‐19 prevalence at the time of the survey was low with new daily cases ranging from 0 to 11 with minimal or no community transmission.^21^ Respondents' median age was 46 years (IQR 35–57), 58% were female, 69% of the cohort had Crohn's disease, 28% had ulcerative colitis, and 2% had IBD‐U. The majority of respondents had their IBD care in the public system (72%), with 23% via the private system and 4% primarily with their GP. Of those with Crohn's disease, some 44 (28%) people had active disease (median HBI 6, IQR 5–7) and 115 people with inactive disease (median HBI 3, IQR 2–4). Of those with ulcerative colitis or IBD‐U, some 16 (24%) people had active disease (median SCCAI 6, IQR 5–8) and 50 people had inactive disease (median SCCAI 2, IQR 1–3).

Current medications and demographics can be seen in Table 1, with 25 (9%) people on no IBD medication. Thiopurines were the most frequently used agents (32%), followed by Infliximab (24%) and Mesalazine (23%). Over half of the cohort was on a biologic medication (56%) with 10 (4%) people on a 5‐ASA and a biologic, 55 (21%) people on an immunomodulator and a biologic, and 7 (3%) on a 5‐aminosalicylates (5‐ASA) and an immunomodulator in addition to a biologic. Of the 12 respondents who were on prednisolone, some 41% had no plan to taper their dose, with most (80%) on a dose less than 20 mg per day.

**Table 1:** Baseline population characteristics including underlying inflammatory bowel disease, and medications by coronavirus disease 2019 pandemic attributed medication change

	*n*	Medication ceased *n*	Medication dose reduced or skipped*n*
Total number of patients	262	8	11
Gender		3	
Female	143	3	7
Male	101		3
Disease			
Ulcerative colitis	71	2	3
Crohn's disease	174	6	8
Indeterminate colitis	6	0	0
Medications			
None	25	1	0
5‐aminosalicylates	70		
Mesalazine	62	1	3
Sulfasalazine	8	0	0
Immunomodulator	100		
Thiopurine	84	1	1
Methotrexate	16	1	2
Biologic	147		
Infliximab	64	2	1
Adalimumab	33	1	1
Ustekinumab	30	0	2
Vedolizumab	20	0	0
Prednisolone	12	2	2
Other	4	0	0
Number of immunosuppressive medications			
None	65	2	3
One	137	5	7
Two	56	1	1
Three	3	0	0

5‐aminosalicylates were not considered immunosuppressive medications. Other medication includes tacrolimus (1), cyclosporine (1), and tofacitinib (2).

Fourteen patients (5%) chose to stop, dose reduce, or omit medications as a direct response to concerns about the COVID‐19 pandemic, with eight patients ceasing a medication and 11 dose reducing or omitting doses. Medications ceased included an immunomodulator in five cases, 5‐ASA in one case, prednisolone in one case, and not detailed in one case. Of those changing their medication due to COVID‐19, four reported discussing this with their IBD physician or nurse, two reported discussion with a complementary therapy provider, and six reported making the decision on their own without consulting their IBD service or general practitioner. Number of immunosuppressive medications was not associated with any medication change due to the COVID‐19 pandemic (*P* = 0.89). Self‐injectable biologic usage was not associated with any medication change due to the COVID‐19 pandemic (*P* = 0.61).

Some 34 (13%) respondents reported starting supplements due to the COVID‐19 pandemic. This included vitamin D (seven instances), vitamin C (six instances), multivitamin (five instances), among other herbs and vitamins. This was on their own advice in the majority of cases (22 instances), but also on the advice of their IBD physician (7 instances), general practitioner (6 instances), naturopath (3 instances), and family or friends (3 instances).

Higher mean disease activity scores were seen in people who reported COVID‐19‐related medication changes compared to those not altering their treatment regime: SCCAI mean (95% confidence interval [CI]) 5.2 (95% CI 2.7–7.8) *versus* 3.6 (3.2–3.9) *P* = 0.043 and HBI mean (95% CI) 4.9 (2.8–6.9) *versus* 3.5 (3.2–3.7) *P* = 0.026. Prednisolone use was associated both with cessation of conventional IBD medication due to the COVID‐19 pandemic (*P* = 0.004) and omission or reduction of doses due to fear of adverse COVID‐19 outcomes (*P* = 0.022).

Examining conventional medication adherence with MARS, 3% of respondents demonstrated low, 20% partial, and 68% satisfactory adherence. Low adherence was associated with any change in medication attributed to the COVID‐19 pandemic (*P* = 0.004), although it was not related to current self‐reported disease activity by HBI or SCCAI (*P* = 0.25, *P* = 0.21).

Any CAM use was reported by 114 respondents (43%), with frequent CAM use reported by 89 respondents (34%). CAM type used can be seen in Table 2 with exercise, probiotics, and meditation most frequently. Stated reasons for using CAM included: mental health (*n* = 64), IBD (*n* = 45), to protect against COVID‐19 (*n* = 6), and other (*n* = 33). The majority of those who used complementary therapies felt that it worked well or very well (59%). Ninety percent of those using CAM continued their IBD medication and over half (58%) disclosed their CAM use to their IBD physician or pharmacist. Frequent complementary therapy usage was associated with low medication adherence scores (*P* = 0.003) but not medication change due to the COVID‐19 pandemic (*P* = 0.89).

**Table 2:** Complementary and alternative medicines (CAMs) use reported by patients with inflammatory bowel disease via an anonymous online survey during the coronavirus disease 2019 pandemic

CAM	Frequency *n* (%)
Exercise	75 (29)
Probiotics	58 (22)
Meditation	35 (13)
Mindfulness	34 (13)
Acupuncture	17 (6)
Prayer	9 (3)
Chinese medicine	8 (3)
Homeopathy	7 (3)
Other	15 (5)

Respondents could choose more than one CAM. Other included vitamin or mineral supplement (4), magnetism (3), turmeric (3), boswellia (1), naturopath (1), cannabis (1), yoga (1), chiropractor (1), and foot reflexology (1).

The BMQ was completed by 232 respondents, with association between BMQ scores, patient medications, disease activity, medication adherence scores, and CAM use in Table 3. The Necessity–Concern differential was higher in those on biologic therapy (*P* = 0.007), indicating greater perceived value of these therapies, which was also associated with satisfactory medication adherence scores (*P* < 0.0001). The Necessity–Concern differential was significantly lower in those with frequent complementary therapy usage (*P* = 0.04) and those with active disease (*P* = 0.008). Patients who had any change to their IBD medication due to COVID‐19 had higher medication Concern scores (*P* = 0.04), but no difference in medication Necessity, Harm, or Overuse scores. Frequent complementary therapy usage was associated with higher Concern score (*P* = 0.021), higher Harm score (*P* = 0.03), and a trend to higher Overuse score (*P* = 0.09).

**Table 3:** Beliefs about medicines questionnaire (ref) outcomes including scores for concern, necessity, necessity–concern differential, harm and overuse by patient medications, complementary therapy usage, medication adherence scores, active disease, and medication change due to coronavirus disease 2019 pandemic

	Concern scoreMean, 95% CI	Necessity scoreMedian, IQR	Necessity–concern differentialMean, 95% CI	Harm scoreMean, 95% CI	Overuse scoreMean, 95% CI
Biologic therapy use (*n* = 148)	14.4 (1.7–15.1) *P* = 0.53	21 (19–24)P < 0.001	6.7 (5.9–7.6) *P* = 0.0038	8.3 (7.8–8.8) *P* = 0.32	9.3 (8.7–9.7) *P* = 0.003
Immunomodulator use (*n* = 99)	14.3 (13.5–15.1) *P* = 0.84	20 (19–23)*P* = 0.37	6.1 (5.0–7.3) *P* = 0.61	8.4 (7.85–9.0) *P* = 0.85	9.6 (8.9–10.3) *P* = 0.73
5ASA therapy use (*n* = 68)	13.7 (12.4–14.9) *P* = 0.20	20 (16–23) *P* = 0.017	5.5 (4.0–7.0) *P* = 0.47	8.1 (7.5–8.7) *P* = 0.28	9.7 (9.0–10.4) *P* = 0.99
Prednisolone usage (*n* = 11)	17 (13.4–20.6) *P* = 0.02	21.5 (20–23)*P* = 0.28	4.4 (0.6–8.1) *P* = 0.37	10.3 (7.4–13.2) P = 0.03	10.8 (8.4–13.2)*P* = 0.26
Frequent complementary therapy usage (*n* = 89)	15.1 (14.2–16.1) *P* = 0.021	20 (18–23)*P* = 0.56	4.9 (3.6–6.2) *P* = 0.04	9 (8.2–9.7) *P* = 0.03	10.2 (9.4–10.1) *P* = 0.09
Adherent medication adherence scores(*n* = 178)	13.7 (13.1–14.3) P = 0.0005	21(19–24)P = 0.0011	6.9 (6.2–7.7) P < 0.0001	8.3 (7.9–8.7) *P* = 0.15	9.3 (8.9–9.8) P = 0.005
Active disease(*n* = 60)	16.1 (15.1–17.1) *P* = 0.0001	20 (18–23)*P* = 0.80	4.2 (2.7–5.7) P = 0.007	9.3 (8.5–10.1) P = 0.007	10.3 (9.4–11.1) *P* = 0.12
Medication change due to COVID‐19 (*n* = 14)	16.6 (13.8–19.4) *P* = 0.04	21(19–22)*P* = 0.99	3.8 (0.62–6.9)*P* = 0.15	9.25 (7.0–11.5)*P* = 0.31	10.4 (8.2–12.7)*P* = 0.37

Necessity–concern differential calculated by taking concern score away from necessity score. Mean and 95% confidence interval (CI) or median and interquartile range (IQR) as appropriate. Comparisons to remaining population using Student's *t*‐test or Wilcoxon rank‐sum test when appropriate. Median necessity score was 20 (IQR 18–23), with mean Concern score of 14.3 (SD 4.2), mean Harm score was 8.4 (SD 2.8), and mean Overuse score was 9.7 (SD 3.1).

## Discussion

In this novel study, examining the influence of the COVID‐19 pandemic on patients' IBD medication adherence and beliefs, rates of suboptimal medication behaviors attributable to the pandemic were relatively low (5%). However, there were important concerning associations with self‐initiated medication changes in this cohort, such as increased prednisolone usage, increased disease activity, and higher medication Concern scores. These findings are alarming, in that most patients did not discuss their concerns or medication changes with their IBD physician, despite this being in a cohort with more severe disease, evidenced by more than half of patients being treated with biologic therapy. The prevalence of COVID‐19 in Australia during the survey was low^21^, which illustrates the significance of the patient concern and nonadherence seen in this study. Areas with higher COVID‐19 prevalence may see higher levels of nonadherence and patient concern along with the observed deleterious associations.

The association between prednisolone usage and COVID‐19 pandemic inspired medication change is consistent with other studies in IBD during the COVID‐19 pandemic with nonadherence to biologic infusion associated with subsequent risk of steroid requirement.^22^ Ironically, emerging data have suggested worsened COVID‐19 outcomes among patients taking prednisolone, yet a negative outcome association between immunomodulators, biologic therapies, and COVID‐19 has not been established.^23^ Interestingly, IBD biologic nonadherence among veterans in one study was 11% in a setting with a much higher burden of COVID‐19.^22^ Of additional concern, active IBD has been found to be associated with worse COVID‐19 outcomes,^4^ rendering long‐term disease control with maintenance therapy all the more important.

It is also noteworthy that a significant proportion of patients commenced supplementary medication as a result of the COVID‐19 pandemic. In addition to this, CAM use was frequent, in line with rates reported in the literature in IBD both at our center and abroad prior to the pandemic.^14^ No association was found between CAM use and pandemic‐related conventional medication change, which may relate to insufficient statistical power. Interestingly, the majority of CAM users cited mental health concerns as the indication for CAM uptake, highlighting an unmet need in IBD care in this cohort.

Different medication beliefs appeared to drive CAM use compared with COVID‐19 pandemic related conventional medication change. Medication modifiers had characteristically higher Concern scores, while CAM users were distinguished by not only higher concerns but also higher perceived Harm and lower perceived Necessity scores. Interestingly, IBD patients taking 5‐ASA demonstrated similar medication Concern scores as those on immunomodulators and biologics, suggesting such concerns may be patient rather than medication specific.

The study population's Necessity and Concern scores were similar to that reported in a study of thiopurine usage in an IBD population,^24^ and a cohort of patients with rheumatoid arthritis,^25^ with both studies prior to the COVID‐19 pandemic. This reflects the significance of the higher Concern scores seen in those within the study population who attributed self‐initiated medication changes to the COVID‐19 pandemic.

The strengths of this study include its multicenter nature, representative age, and gender distribution, especially high proportion of biologic patients and use of validated scores for medication adherence and beliefs about medicines. Limitations include selection bias, with the study population likely including more engaged and adherent patients and potentially underrepresenting medication under adherence. It is also possible that the respondent populations' interest in the study reflects concern over the COVID‐19 pandemic consequently leading to higher medication Concern scores. Other COVID‐19 risk factors that may have influenced patients' decision‐making, such as obesity or cardio‐respiratory comorbidities, were beyond the scope of this survey. The response rate to the survey was reflective of the use of unsolicited email invitations to the survey and the anonymous online format of the survey.

In conclusion, in a large tertiary IBD population in the setting of low COVID‐19 prevalence, the pandemic influenced IBD medication adherence negatively in 1 in 20 patients. This nonadherence was associated with deleterious outcomes such as increased disease activity and corticosteroid use, as well as higher perceived concerns regarding IBD medication. Individuals using CAM cited more concerns and more perceived harm from conventional medication and were more likely to report medication nonadherence. These findings emphasize the critical role of a strong doctor–patient relationship to address medication concerns, and the provision of updated information to consumers as further evidence guiding the use of IBD medications during the COVID 19 pandemic emerges.

## Supporting information


**Appendix S1.** Covid 19 Questions.

**Appendix S2.** IBD History.
