# Supplementary material for: Medication adherence and complementary therapy usage in inflammatory bowel disease patients during the coronavirus disease 2019 pandemic
Source: JGH Open. 2021 Mar 29;5(5):585–9. doi: 10.1002/jgh3.12537 (PMC8114986; doi:10.1002/jgh3.12537)
Supplement: Supplementary file 1 — Appendix S1. Covid 19 Questions. [file JGH3-5-585-s001.pdf]

Covid-19 Questions

The following questions are about the medication you are taking for inflammatory bowel disease during the coronavirus (COVID19) pandemic.

Have you changed your inflammatory bowel disease medication as a result of the coronavirus pandemic?	◯ Yes<br>◯ No
If you have, please tell us what medications you have changed:	__________________________
Have you reduced the dose or skipped doses of your inflammatory bowel disease medication as a result of the coronavirus pandemic?	◯ Yes<br>◯ No
If yes, please tell us what medication you skipped and how did it:	__________________________
If you decided to stop or reduce your medication for your inflammatory bowel dsiease on what information did you base this on?	☐ GP<br>☐ IBD doctor advice<br>☐ IBD nurse advice<br>☐ Naturopath advice<br>☐ Other complementary or alternative practitioner advice<br>☐ Crohn's colitis Australia provided information<br>☐ Information provided by your hospital IBD service<br>☐ Friends or family<br>☐ Other
If other, please tell us:	__________________________
Has your doctor changed the dose interval of your infusion or subcutaneous biologic medication for your inflammatory bowel disease? (for example, infliximab to be given every 10 weeks rather than every 8 weeks)	◯ Yes<br>◯ No
If so, please describe your new dosage interval	__________________________
Have you started any other medication as a result of the coronavirus pandemic?	◯ Yes<br>◯ No
If yes, please provide detail:	__________________________
Have you started taking any additional supplements (e.g. vitamin D), or herbal remedies as a result of the coronavirus pandemic?	◯ Yes<br>◯ No
If yes, please provide detail:	__________________________

If yes, on whose advice have you started these supplements or herbal remedies?	☐ Self<br>☐ Internet<br>☐ Family / friends<br>☐ GP<br>☐ IBD specialist<br>☐ Naturopath / Herbal medicine specialist<br>☐ Other
If other, please tells us what:	__________________________________
