# Supplementary material for: Medication adherence and complementary therapy usage in inflammatory bowel disease patients during the coronavirus disease 2019 pandemic
Source: JGH Open. 2021 Mar 29;5(5):585–9. doi: 10.1002/jgh3.12537 (PMC8114986; doi:10.1002/jgh3.12537)
Supplement: Supplementary file 2 — Appendix S2. IBD History. [file JGH3-5-585-s002.pdf]

IBD history

Please enter your age.	__________________________________
What is your gender?	◯ Male<br>◯ Female<br>◯ Other
In which system does the majority of your care of your inflammatory bowel disease take place?	◯ Public<br>◯ Private<br>◯ GP<br>◯ Other
If other please tell us where:	__________________________________
What type of inflammatory bowel disease do you have?	◯ Ulcerative colitis<br>◯ Crohn's disease<br>◯ Indeterminate colitis
Please tick any medications you are currently on for your inflammatory bowel disease?	☐ Mesalazine<br>☐ Sulfasalazine<br>☐ Azathioprine<br>☐ Mercaptopurine<br>☐ Methotrexate<br>☐ Tacrolimus<br>☐ Cyclosporine<br>☐ Adalimumab (Humira)<br>☐ Infliximab (Remicade)<br>☐ Ustekininumab (Stelara)<br>☐ Vedolizumab (Entyvio)<br>☐ Golimumab (Simponi)<br>☐ Tofacitinib (Xeljanz)<br>☐ Budesonide (Cortiment, Entocort, Budenofalk)<br>☐ Vitamin D<br>☐ Batrim<br>☐ Prednisolone
If you are on prednisolone - what dose are you currently taking?	__________________________________
If you are on prednisolone - is there a scheduled taper planned?	◯ Yes<br>◯ No
Are you currently taking a clinical trial drug for inflammatory bowel disease?	◯ Yes<br>◯ No
If you are taking a clinical trial drug can you tell us what the name of the drug is?	__________________________________
